# Conceptual Knowledge Shapes the Neural Representations of Learned Faces in Non-Visual Regions of the Brain

**DOI:** 10.1523/JNEUROSCI.0122-25.2025

**Published:** 2025-07-07

**Authors:** Kira N. Noad, David M. Watson, Timothy J. Andrews

**Affiliations:** Department of Psychology and York Neuroimaging Centre, University of York, York YO10 5DD, United Kingdom

**Keywords:** face, memory, naturalistic, recognition

## Abstract

When we encounter people in real life, increased visual experience with their face is accompanied by an accumulation of conceptual knowledge about them. This conceptual knowledge has been shown to play an important role in face recognition. However, the extent to which conceptual knowledge influences neural responses to faces in visual or non-visual regions of the brain is not clear. To address this question, participants (male and female) learned faces in a naturalistic viewing paradigm in which conceptual information was modulated by presenting a movie to participants in either its original sequence or a scrambled sequence. Although participants in both groups had the same overall perceptual experience, this manipulation had a significant effect on the conceptual understanding of events. After a delay, participants viewed a new movie featuring the previously learned faces while neural activity was measured using fMRI. No significant differences were observed between the Original and Scrambled groups in core face-selective regions of the visual brain. This aligns with the fact that overall exposure to faces was consistent across groups, ensuring that our manipulation did not impact visual processing of faces. In contrast, differences between the groups were evident within a network of regions that are typically associated with processing person knowledge. This network of regions was also able to discriminate the identity of the key characters based on the response to the faces. These findings provide important insights into the level of neural processing at which conceptual knowledge influences familiar face recognition during natural viewing.

## Significance Statement

The ability to recognize faces relies on the depth of processing during encoding, not just perceptual exposure. Associating faces with conceptual information enhances recognition by fostering deeper processing. However, the neural level at which this occurs remains unclear. One possibility is that conceptual information strengthens visual representations; alternatively, it may directly influence non-visual regions involved in processing person knowledge. Using a naturalistic movie-viewing paradigm, this study found consistent neural responses in visual regions regardless of conceptual coherence. However, a network linked to person recognition showed significant effects of conceptual knowledge. These findings provide new insights into how real-world learning integrates perceptual and conceptual information, enriching our understanding of the neural mechanisms underlying face recognition.

## Introduction

Recognizing familiar faces is a fundamental cognitive function that plays a pivotal role in social interaction and communication. However, the process of recognizing a face under natural viewing conditions poses considerable computational challenges for the brain (Young and Burton, 2017). Nonetheless, most humans demonstrate a remarkable ability to recognize the faces of people that they are familiar with (Bruce, 1982; Burton, 2013). In contrast, the task of matching unfamiliar faces is prone to errors, even when the image undergoes relatively minor changes (Hancock et al., 2000). Cognitive models of face perception propose that the process of familiarization with a face involves the development of image-invariant visual representations (Bruce and Young, 1986; Young and Burton, 2017). During the familiarization process, it is hypothesized that these image-invariant representations emerge through repeated exposure to different visual instances of a person's face (Burton et al., 2011; Kramer et al., 2018). Empirical evidence supports this theoretical framework, demonstrating that increased visual exposure improves recognition performance (Murphy et al., 2015; Ritchie and Burton, 2017; Juncu et al., 2020).

In real life, increased perceptual exposure to a face is accompanied by a corresponding increase in conceptual information about the individual. This conceptual information includes knowledge about the person and the nature of our relationship with them. Emerging evidence suggests that conceptual knowledge may also play critical role in becoming familiar with a face. Several studies have demonstrated that recognition accuracy improves when participants engage in semantic judgements while learning new faces, as opposed to purely visual tasks (Bower and Karlin, 1974; Patterson and Baddeley, 1977; Schwartz and Yovel, 2016, 2019). Furthermore, face recognition is facilitated when a familiar face is preceded by another face associated with similar conceptual knowledge (Bruce and Valentine, 1986).

Previous studies investigating the role of conceptual knowledge in face recognition have largely focused on the response to static faces paired with arbitrary conceptual knowledge. However, in everyday situations, faces are encountered within dynamic and context-rich scenes, where conceptual information about individuals is integrated within an evolving narrative (Redcay and Moraczewski, 2020; Jääskeläinen et al., 2021). To bridge this gap, we previously developed a naturalistic viewing paradigm in which participants viewed a movie, either in its original sequence or in a scrambled sequence (Noad and Andrews, 2024). Both groups were exposed to the same overall visual content, but conceptual coherence was maintained only in the original sequence. Nevertheless, participants who viewed the original sequence developed better recognition of the faces, which was still evident after a delay of a few weeks. This suggests that conceptual knowledge may play an important role in familiar face recognition.

The aim of this study was to explore how conceptual knowledge shapes the neural responses to newly learned faces using the naturalistic viewing paradigm. Participants viewed the encoding movie in its original sequence or in a scrambled sequence. After a period of memory consolidation (Noad and Andrews, 2024), we measured neural responses, using functional magnetic resonance imaging (fMRI), while participants viewed a “recognition” movie featuring the faces of the main characters. Our goal was to determine the level of processing at which conceptual knowledge affects the neural representation of faces. One possibility is that conceptual knowledge could enhance the perceptual processing of stimuli, which in our paradigm would lead to more robust representations of learned faces within the core face-selective regions of the visual cortex. Alternatively, conceptual knowledge could modulate activity in non-sensory regions that are directly associated with person knowledge related to a face. The results show that, although the neural response in visual regions was similar across groups, a network of non-visual regions associated with person knowledge displayed significant effects of conceptual knowledge. These findings offer critical insights into the neural mechanisms by which conceptual knowledge impacts the level of processing involved in familiar face recognition.

## Materials and Methods

### Participants

We recruited participants into two groups: (1) participants who watched the encoding movie in the Original sequence and (2) participants who watched the encoding movie in a Scrambled order.

A total of 38 participants took part in this study, with 19 participants randomly assigned to the Original condition (median age, 20 years; age range, 18–31, 7 male) and 19 to the Scrambled condition (median age, 20 years; age range, 18–27, 6 male). All participants were neurologically healthy (as indicated by self-report), were right-handed, and had normal or corrected-to-normal vision. None of the participants had prior familiarity with the TV show *Life on Mars*, which was used as the stimulus in this study. To ensure that participants had normal face recognition abilities, each individual completed the Cambridge Face Memory Test (CFMT, Duchaine and Nakayama, 2006). A score of 65% or above indicates typical face recognition abilities. All participants scored above this threshold on the CFMT except one participant scoring just below (58.3%). The mean CFMT score for the sample was 79.9% (standard deviation, 9.3%). The sample size was determined a priori based on previous fMRI studies using naturalistic stimuli and similar analysis methodologies (Hasson et al., 2008, 2009; Chen et al., 2017; Andrews et al., 2019). Written informed consent was obtained from all participants and the study protocol was approved by the York Neuroimaging Centre Ethics Committee.

### Encoding movie

**Figure 1.**
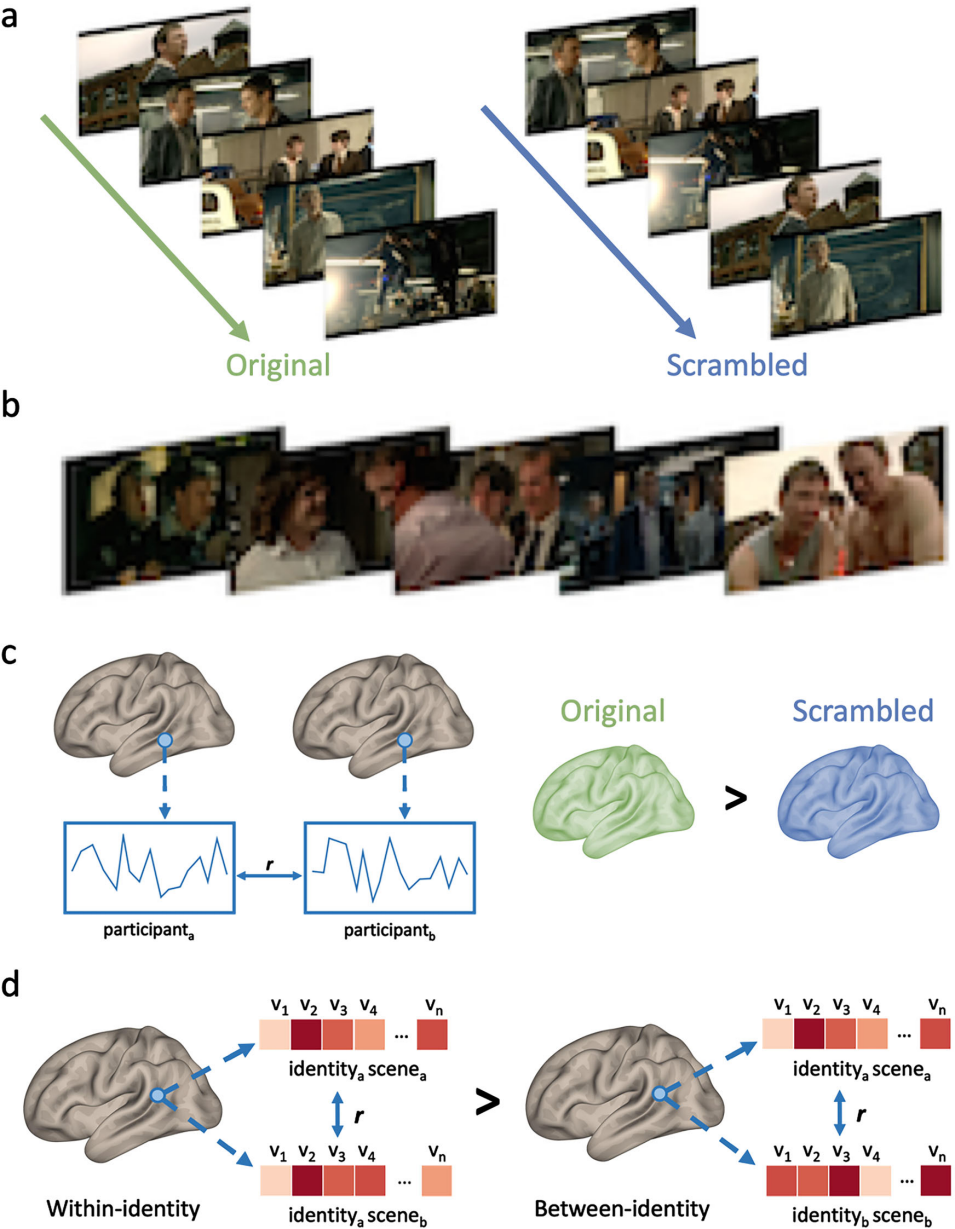
Study design and neuroimaging analysis. ***a***, During the encoding phase, participants watched an encoding movie from TV show *Life on Mars*, presented in its Original order or a Scrambled order. Both conditions provided identical visual exposure to the faces of the characters, but the Scrambled condition impaired the ability to integrate the associated conceptual knowledge due to the disordered narrative. ***b***, In the recognition phase, all participants watched a movie comprising clips from unseen episodes of *Life on Mars*, while brain activity was measured using fMRI. The clips featured the same characters shown during the learning phase. ***c***, Neural responses were analyzed across individuals using intersubject correlation (ISC, left), in which the time-course of voxel responses were correlated (*r*) between individuals. Differences in ISC values were subsequently compared between the Original and Scrambled groups (right). ***d***, Patterns of neural response to specific identities in the movie were analyzed using multivoxel pattern analysis within each region of interest. The voxelwise pattern of response to the same identity (within-identity) or to different identities (between-identity) were correlated across different scenes. Within-identity correlations were compared with between-identity correlations to identify brain regions showing an identity-specific pattern of response.

Approximately 4 weeks before the fMRI scan session, participants viewed an encoding movie outside of the scanner. The specific version of the movie they watched depended on their assigned experimental condition: (1) Original or (2) Scrambled group (Fig. 1*a*). Both versions of the movie were 20 min in duration (1,170 s) and were constructed from audiovisual clips from the first episode of the BBC TV series *Life on Mars*, a British police drama that follows a police officer who travels back in time after being involved in a road accident. It was chosen because actors were likely to be unfamiliar to the participant pool and because it contains a detailed and rich narrative which would be very difficult to follow when viewed in a scrambled order. In the Original condition, the clips were presented in the original order, preserving the coherent narrative structure of the episode. A total of 14 clips were used, with an average duration of 84 s (range 39–228 s). Conversely, in the Scrambled condition, the same clips were presented in a randomized order, disrupting the coherence of the narrative. The clips were assigned a random order for the Scrambled condition, with longer clips subdivided into shorter segments, resulting in a mean clip length of 39 s. All participants in the Scrambled condition viewed the same random order of clips. Importantly, the overall visual input remained identical between both conditions, ensuring that participants in both were exposed to the same content, but with varying narrative coherence.

### Conceptual knowledge

After viewing the encoding movie, participants underwent an assessment to evaluate their conceptual understanding of the presented clips. The assessment included both a free recall task, in which participants were instructed to describe the stimulus in as much detail as possible and a set of eight structured questions targeting specific events in the video. Each question was accompanied by a static image representing the relevant event. Performance on these tests was independently evaluated by two raters (KNN, GS), who were blind to the experimental condition, using a predefined scoring scheme.

The free recall test was evaluated based on 10 key events that occurred during the encoding video. Raters assigned scores of 0, 1, or 2 for each event, depending on whether the participant provided no description, a partial description, or a full description of the event, giving a maximum possible score of 20. Similarly, the eight structured questions were scored using the same 0–2 scale, allowing for a maximum score of 16. Inter-rater reliability was evaluated using intra-class correlation coefficient (ICC) with a two-way mixed model and Agreement definition. Excellent agreement was found between raters for the free recall test, with an ICC of 0.93 and 95% confidence intervals of 0.87–0.96 (*F*_(37,37.8)_ = 26.7, *p* < 0.001). The structured question test also demonstrated strong reliability, with an ICC of 0.94 and 95% confidence intervals of 0.89–0.97 (*F*_(37,37.8)_ = 32.1, *p* < 0.001). Subsequent analyses were based on the average scores across raters.

### Face recognition

Following the encoding phase, participants completed a recognition memory task involving actors featured in the video clips. Faces were presented individually in a randomized order and remained on screen until a response was made. Participants were instructed to press a button to indicate if the identity of the face corresponded to any of the actors observed in the previously viewed video. The test included static faces of 10 actors from the video, which were extracted from the *Life on Mars* TV series but did not correspond to the exact frames shown in encoding video. Another face from each actor, taken from outside of the *Life on Mars* TV series, was included in the test. For each target actor, two foil images of different individuals were selected to match age, expression, hairstyle, lighting, and overall appearance. Sensitivity (*d*’) was calculated for each participant using hit rates (i.e., correct recognition of faces as being present in the movie) and false alarm rates (i.e., incorrect identification of foils as present in the movie). Data for the recognition memory task was unavailable for two participants in the Original condition.

### Recognition movie

Approximately 4 weeks after viewing the encoding movie (mean, 31.3 d; range, 23–41 d), participants watched a new movie containing previously unseen clips from the first season of *Life on Mars* while neural activity was recorded using fMRI (Fig. 1*b*). The rationale for implementing a delay of 4 weeks was to investigate the enduring effects of narrative structure on recognition memory. This delay allowed us to be consistent with a previous behavioral study, in which we showed that differences in face recognition between participants who viewed original or scrambled versions of the encoding video persisted after a similar 4 week interval (Noad and Andrews, 2024). The recognition movie focused on five main characters from the TV series. The clips were selected from different episodes and, as such, did not form a coherent narrative. The movie was projected onto an in-bore screen at a distance of 57 cm from the participant with the image subtending approximately 38.7 × 22.3° of visual angle. Audio accompanying the movie was played to participants during the scan. The movie was a total of 12 min 46 s, composed of 11 distinct scenes ranging in length from 37 to 119 s. The movie was presented using PsychoPy (Peirce et al., 2019).

### fMRI data acquisition

Scanning was conducted using a 3 T Siemens Magnetom Prisma MRI scanner equipped with a 64-channel phased array head coil at York Neuroimaging Centre. Functional data were acquired using a gradient-echo echoplanar imaging (EPI) sequence from 60 axial slices (TR, 2 s; TE, 30 ms; FOV, 240 × 240 mm; matrix size, 80 × 80; voxel dimensions, 3 × 3 × 3 mm; slice thickness, 3 mm; flip angle, 80°; phase encoding direction, anterior to posterior; multiband acceleration factor, 2). Additionally, T1-weighted structural images were acquired from 176 sagittal slices (TR, 2,300 ms; TE, 2.26 ms; matrix size, 256 × 256; voxel dimensions, 1 × 1 × 1 mm; slice thickness, 1 mm; flip angle, 8°). Field maps were collected from 60 slices (TR, 554 ms; short TE, 4.90 ms; long TE, 7.38 ms; matrix size, 80 × 80; voxel dimensions, 3 × 3 × 3 mm; slice thickness, 3 mm; flip angle, 60°).

fMRI data were analyzed using FSL's FEAT v6.0 (http://www.fmrib.ox.ac.uk/fsl; Jenkinson et al., 2012) Preprocessing steps included motion correction using MCFLIRT (Jenkinson et al., 2002), temporal high-pass filtering (Gaussian-weighted least squares straight line fittings, sigma = 50 s), and slice timing correction. Spatial smoothing was applied with a 6 mm FWHM Gaussian kernel. Non-brain material was removed using the Brain Extraction Tool (BET; Smith, 2002). Functional data were registered to a high-resolution T1-anatomical image via boundary-based registration (Greve and Fischl, 2009) and subsequently normalized to the standard MNI152 brain template using non-linear registration computed with FNIRT (Andersson et al., 2010). Field maps were incorporated to correct for distortions in functional images during the registration step.

### Intersubject correlation

To examine brain regions that reflected group differences in conceptual information processing, we measured intersubject correlations (ISC) in neural response across participants within each group during the Recognition Movie. To compute the ISCs, time series from each voxel were converted to percent signal change, and six head motion parameters were regressed out. The resulting time series were then correlated (Pearson's *r*) with corresponding voxels from other participants in the same group, using a leave-one-out approach. Specifically, for each participant, the time series of each voxel was correlated with the average time series of the group (*N*–1) in each corresponding voxel. A Fisher's *z* transform was applied to the correlations.

To compare the Original and Scrambled groups, a permutation test (BrainIAK; Kumar et al., 2021) was used to assess significance of group differences in the ISCs by randomizing the group assignment of participants 10,000 times to create a null distribution. From this, whole-brain *p* statistic maps were created for the contrast of Original–Scrambled, which we represented in negative log units. A cluster correction for multiple comparisons was then applied to these maps using an initial cluster forming threshold of −log_10_(*p*) > 2 (*p* < 0.01) and a cluster significance threshold of *p* < 0.05.

### Multivoxel pattern analysis

To investigate whether specific brain regions exhibited identity-specific patterns of activity, we compared the pattern of neural response to faces from five characters (Sam, Gene, Ray, Chris and Annie) that featured in the recognition movie (Fig. 1*d*). The movie stimulus was divided into 14 scenes (7 odd, 7 even). The occurrences of the main characters were tagged in the odd and even scenes. For a character to be tagged, it had to include a clear image of the face that was present for at least 2 s. To ensure accuracy, two independent raters (TJA, GW) conducted the tagging prior to resolving any discrepancies in timing. The tagging facilitated the creation of separate regressors for each character in the odd scenes and another set for the even scenes. The regressors were boxcars on the onscreen appearance convolved with a double gamma HRF. These regressors, along with their temporal derivatives and head motion regressors, were then entered into a first-level GLM analysis (Woolrich et al., 2001). This analysis generated 10 parameter estimates for each participant, which were subsequently normalized by subtracting the voxelwise mean of the estimates for odd and even scenes independently and transforming them into MNI space.

These parameter estimates were then used for multivoxel pattern analysis (MVPA). For each region of interest (ROI), Pearson's correlation coefficients were calculated between the patterns of the parameter estimates for the same versus different identities. These comparisons were always performed between odd and even scenes. Given that the other characters did not feature prominently in the encoding movie, we focused primarily on the two main characters (Sam and Gene). Correlations were performed on the patterns from the core face regions (defined from a localizer scan) and the extended network region (defined from the ISC analysis). The two within-identity correlations (Sam-odd vs Sam-even and Gene odd vs Gene even) were compared against 16 between-identity correlations (odd-even correlations of both main characters with each of the other characters across odd and even scenes). Within- and between-identity correlations were Fisher's *z* transformed and averaged for each participant. To test for identity-specific patterns of activity, the within- and between-identity correlations were compared using paired sample *t* tests within each group. Greater correlations for within-identity comparisons relative to between-identity comparisons would indicate a significant identity-specific response.

### Localizer scan

A localizer scan was conducted to delineate face-selective and scene-selective regions of interest. The scan comprised three stimulus conditions: faces, scenes, and phase scrambled faces. Face stimuli were presented in three different viewpoints (−45, 0, 45°) and were sourced from the Radboud database of face stimuli (Langner et al., 2010). Faces were displayed against a 1/f amplitude-mask background. Scrambled faces were created by randomizing the phase spectra while maintaining the amplitude spectra of the face images including the amplitude mask background. Scene stimuli included indoor and outdoor images sourced from the SUN database (Xiao et al., 2010). Each image subtended approximately 8.4 × 8.4° of visual angle. Four images from each condition were presented in each block for 600 ms with a 200 ms ISI, followed by a 6 s blank period, for a total of 9 s per block. Nine blocks were presented for each condition in a pseudorandomized order, for a total scan time of 244 s. To ensure participant engagement, they were required to respond via button press whenever a change in color of the fixation cross occurred.

Boxcar models of each stimulus block were convolved with a single-gamma hemodynamic response function to create regressors for each condition. These were then incorporated into a first-level GLM analysis (Woolrich et al., 2001) alongside their temporal derivatives and confound regressors for six head motion parameters. Individual participant data were subsequently entered into a higher-level group analysis using a mixed-effects GLM using FLAME (Woolrich et al., 2004). Face-selective regions were identified through contrasts of the response to faces against the other conditions (faces > scenes + scrambled face). ROIs were defined using a clustering algorithm that iteratively adjusted the statistical threshold to grow clusters of 250 spatially contiguous voxels (2,000 mm^3^) around seed voxels within each region. The fusiform face area (FFA), occipital face area (OFA), and superior temporal sulcus (STS) were defined as face-selective regions.

## Results

### Conceptual knowledge

**Figure 2.**
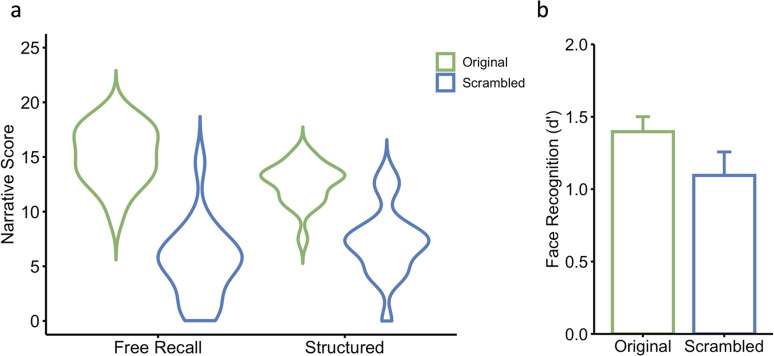
***a***, Greater narrative scores on both measures of conceptual understanding were found for participants in the Original group compared with the Scrambled group. ***b***, Greater recognition of the faces from the movie was found for participants in the Original group compared with the Scrambled group. Error bars denote standard errors.

We first investigated whether manipulating the order of events in the encoding phase would affect conceptual knowledge. To assess this, we compared narrative scores between the Original and Scrambled groups on the free recall and structured question tasks. Consistent with our prior findings (Noad and Andrews, 2024), the Original group demonstrated significantly higher scores on both the free recall task (*t*_(33.8)_ = 10.0, *p* < 0.001, Cohen's *d* = 3.24) and structured question task (*t*_(29.2)_ = 6.3, *p* < 0.001, Cohen's *d* = 2.05; Fig. 2*a*). These findings suggest that presenting events in their original coherent order fosters a deeper understanding of conceptual knowledge.

### Face recognition

In a previous study, we demonstrated that the level of conceptual knowledge influences face recognition, with higher accuracy observed in the Original condition (Noad and Andrews, 2024). In the current study, we again compared behavioral face recognition scores on the faces from the movie between the Original and Scrambled groups. While participants in the Original group exhibited higher face recognition scores than those in the Scrambled group (Fig. 2*b*), this difference did not reach statistical significance (*t*_(30.1)_ = 1.57, *p* = 0.064, Cohen's *d* = 0.51). Nevertheless, the trend was in the expected direction and the effect size was comparable (current study: Cohen's *d* = 0.51; Noad and Andrews, 2024: Cohen's *d* = 0.33). The difference between studies presumably reflects a difference in sample size (current study: *n* = 38; Noad and Andrews, 2024: *n* = 200).

### Intersubject correlation

**Figure 3.**
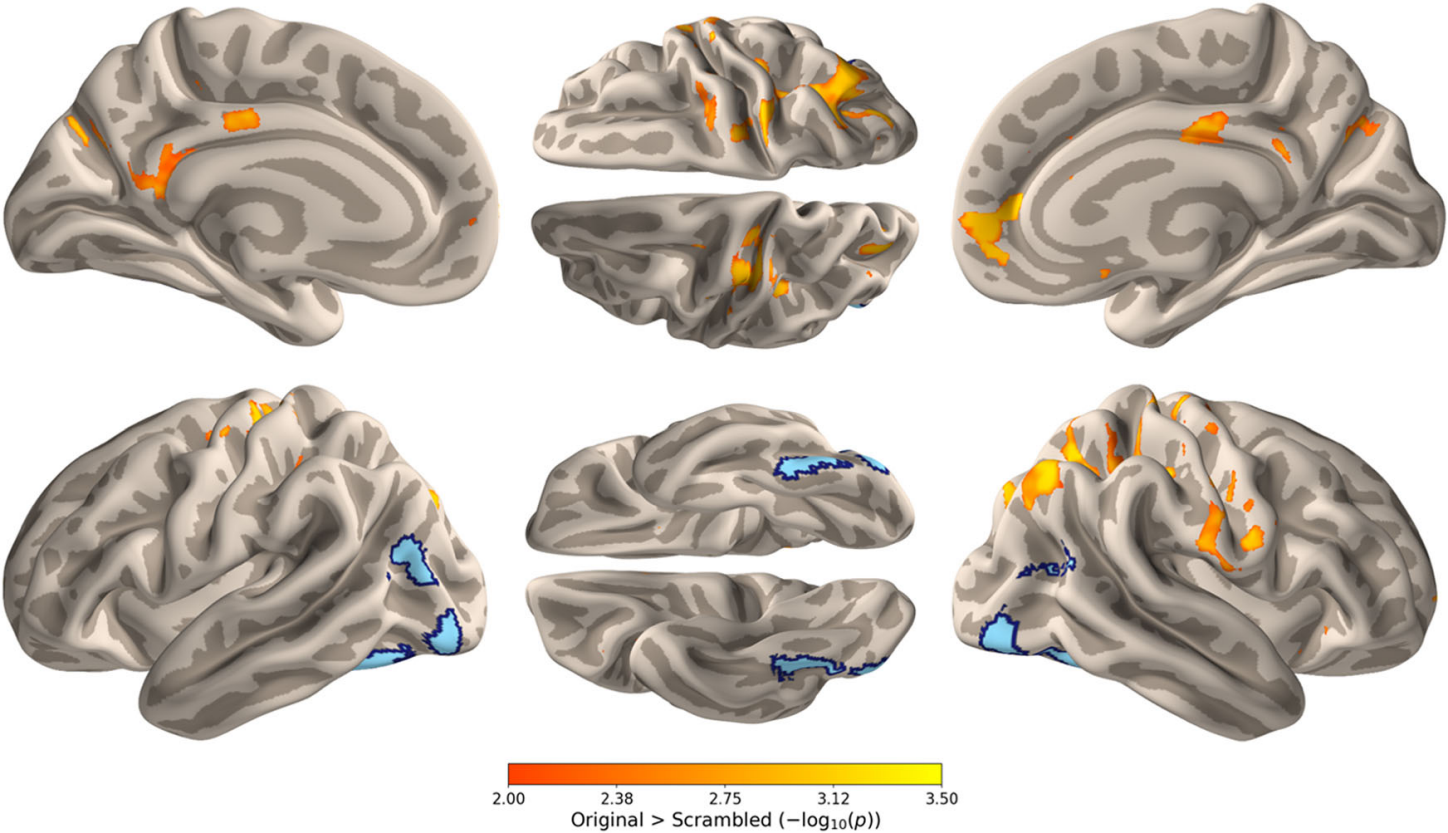
(Top) ISC comparison between participants in the Original and Scrambled groups when viewing a movie containing the faces of the main characters. Voxels in temporal, parietal, and frontal cortex showed significantly higher ISC for the Original group (red-yellow; cluster corrected). No voxels showed significantly higher ISCs for the Scrambled group. (Bottom) The core face-selective regions (blue) were defined by an independent localizer scan. There was no overlap between the core face-selective regions and the regions from the ISC analysis.

To investigate the level of processing at which conceptual knowledge influences face recognition, we examined intersubject correlations (ISC) of neural responses while participants viewed a previously unseen movie containing clips that prominently featured the faces of the main characters. We compared ISCs between participants in the Original and Scrambled groups to assess how narrative coherence during encoding influences neuronal responses to faces during recognition. Regions showing significantly higher ISCs in the Original group were evident across the temporal, parietal, and frontal lobes in both hemispheres (red-yellow; Fig. 3, Table 1). These areas included key regions of the extended face network, such as the amygdala, insula, precuneus, medial prefrontal cortex, and the temporal-parietal junction (Gobbini and Haxby, 2007). In contrast, no significant ISC differences were found for the Scrambled group compared with the Original group, suggesting that the scrambled narrative did not enhance the synchronization of the face processing across subjects. The location of the core face-selective regions (blue) defined by an independent localizer scan shows that there was no overlap between face-selective regions and those regions showing higher ISC in the Original group (Fig. 3, Table 2). This indicates that the enhanced ISC associated with conceptual knowledge involves a broader network beyond the core face-selective regions.

**Table 1.** Regions showing significantly greater ISCs for participants in the Original group compared with the Scrambled group

Region	Hemisphere	*x*	*y*	*z*	max −log_10_(*p*)	Size
Accumbens	L	−11	17	−3	2.16	109
	R	10	12	−8	2.30	102
Amygdala	L	−21	0	−16	2.10	102
Anterior cingulate	R	16	22	30	2.40	100
Frontal pole	L	−14	68	8	2.00	98
	R	18	68	2	2.40	98
Insula	R	26	15	−11	2.00	93
Mid cingulate	L	−2	−20	37	2.00	91
	R	6	−11	30	2.70	148
Medial prefrontal cortex	L	−15	51	−7	2.00	88
	R	9	53	3	3.00	263
Posterior cingulate	L	−9	−47	20	2.52	92
	R	5	−42	23	2.40	102
Postcentral gyrus	L	−31	−26	59	3.00	244
	R	26	−29	71	2.70	109
Postcentral gyrus 2	R	66	−9	18	2.70	100
Precentral gyrus	L	−20	−24	64	2.70	218
	R	22	−23	72	2.70	105
Precentral gyrus 2	R	60	1	24	2.30	111
Precuneus	L	−11	−66	31	2.40	104
	R	14	−62	35	2.52	78
Precuneus 2	L	−21	−78	35	3.00	95
	R	26	−78	35	2.70	117
Superior frontal gyrus	L	−19	−9	57	2.00	36
	R	28	−11	60	2.52	105
Superior parietal lobule	R	27	−54	48	2.70	175
Supramarginal	L	−34	−37	39	3.00	98
	R	39	−27	38	3.00	124
Supramarginal 2	R	46	−29	48	2.52	149
Temporal parietal junction	L	−24	−74	49	2.40	100
	R	41	−63	48	3.00	367

MNI mm coordinates and the corresponding statistical value are provided for the peak voxel in each cluster.

**Table 2.** MNI mm coordinates of maximum voxel of face-selective regions (OFA, occipital face area; FFA, fusiform face area; STS, superior temporal sulcus) defined by an independent localizer scan (faces > scrambled faces + scenes) with maximum *z* score from the localizer contrast and percent overlap of these ROIs with the regions found in the ISC analysis

Region	Hemisphere	*x*	*y*	*z*	Mask size (voxels)	Threshold (*z*)	Overlap (%)
OFA	L	−42	−84	−10	250	5.71	0
	R	46	−78	−6	250	5.94	0
FFA	L	−42	−48	−22	251	5.05	0
	R	42	−52	−18	250	5.29	0
STS	L	−48	−72	18	250	3.98	0
	R	58	−62	16	249	4.17	0

A potential explanation for the lack of overlap between the ISC analysis and the core face regions is that ISC may be inherently lower in these regions, potentially due to variability in eye gaze patterns across participants. Such variability could disrupt temporal synchrony in regions that are strongly driven by the visual input. To evaluate this possibility, we quantified baseline ISC (collapsed across experimental groups) in early visual cortex, core face regions, and extended face regions. If lower ISC in the core network were attributable to increased variability in visual input, we would expect correspondingly low ISC in visual regions. A one-way ANOVA shows that ISCs differ between regions, but not in this direction (*F*_(2.1,77.9)_ = 333.3, *p* < 0.001, *η*^2^ = 0.90). ISCs were highest in the EVC compared with core (*t*_(37)_ = 5.25, *p* < 0.001) and extended face regions (*t*_(37)_ = 24.5, *p* < 0.001). Furthermore, ISC in the core face regions significantly exceeded that of the extended face network (*t*_(37)_ = 19.3, *p* < 0.001). These results indicate that the reduced overlap observed in the core face regions between the Original and Scrambled conditions is unlikely to stem from increased variability in visual input.

### MVPA

We next examined whether patterns of neural activity could discriminate between the faces of the main characters from *Life on Mars*. The occurrence of each of the five main characters was tagged across all scenes in the recognition movie (Milivojevic et al., 2016; Lally et al., 2023). The response to each identity was then measured independently across odd and even scenes. To assess identity-specific neural representations, we computed correlations between patterns of response to the same identity (within-identity) across odd and even scenes and compared them to correlations between responses to different identities (between-identity) across odd and even scenes. The within-identity comparisons were restricted to the two main characters (Sam and Gene) as only they appeared prominently in the encoding movie. This analysis was conducted in the core face regions and in the extended network of regions revealed in the ISC analysis. An identity-specific representation would be evident if within-identity correlations are significantly higher than between-identity correlations. To test for the presence of identity-specific patterns, we performed a 2 × 2 × 2 mixed-design ANOVA including within-subjects factors for Identity (within, between) and Region (core, extended) and a between-subjects factor for Group (original, scrambled). A significant main effect of Identity was found, with within-identity correlations significantly greater than between-identity correlations (within-identity *M*: 0.13, SE: 0.03; between-identity *M*: −0.07, SE: 0.01), demonstrating robust identity-specific representations (*F*_(1,36)_ = 29.10, *p* < 0.001, *η*^2^ = 0.45). There was also a significant main effect of Region (*F*_(1,36)_ = 19.50, *p* < 0.001, *η*^2^ = 0.35), with higher correlations in the extended network (*M* = 0.08, SE = 0.02) compared with core face regions (*M* = −0.01, SE = 0.02). All interactions and main effect of Group were non-significant (*p* < 0.05).

**Figure 4.**
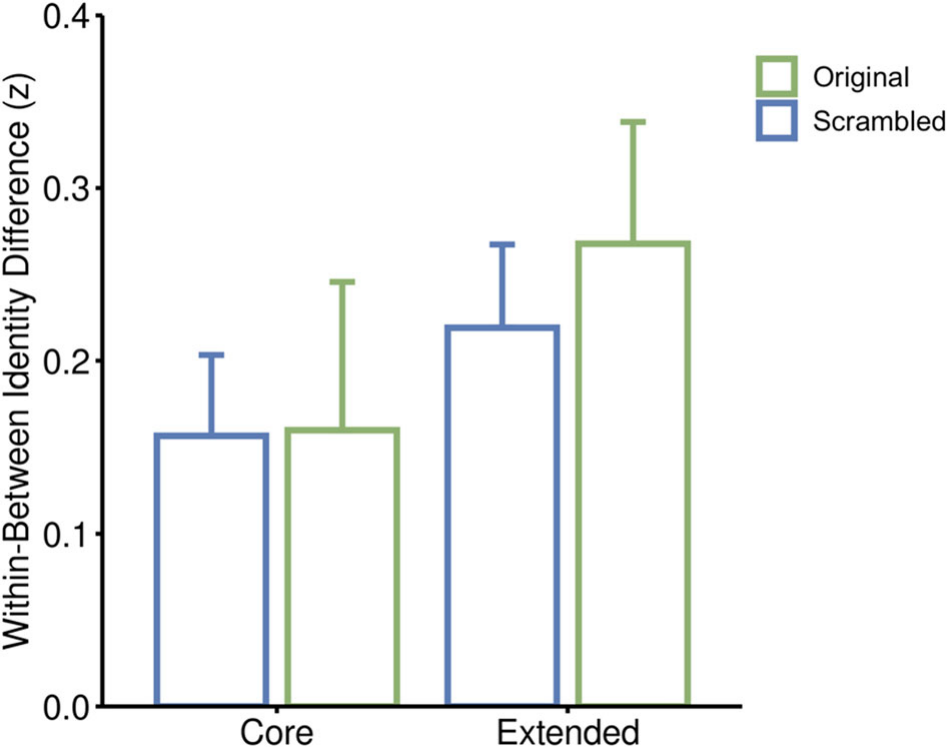
Identity-specific neural patterns for the Original and Scrambled groups in core face regions and the extended network found in the ISC analysis. Identity patterns were greater in the extended regions, although this is not significantly different. Error bars denote standard errors.

To determine whether the identity effects were modulated by conceptual knowledge, we conducted a Group (original, scrambled) × Region (core, extended) mixed-design ANOVA on the within-identity minus between-identity differences for participants in the Original and Scrambled groups. Although the extended regions exhibited a larger within-between identity difference compared with the core regions (Fig. 4), the main effect of region was not significant (*F*_(1,36)_ = 2.45, *p* = 0.120, *η*^2^ = 0.07). Similarly, there was no significant main effect of Group (*F*_(1,36)_ = 0.10, *p* = 0.730, *η*^2^ < 0.01) or Group × Region interaction (*F*_(1,36)_ = 0.20, *p* = 0.673, *η*^2^ < 0.01).

We then examined the effect of identity comparing within-identity and between identity correlations across individual regions within the core face-selective network (Table 3) and regions of the extended network identified from the ISC analysis (Table 4) for both the Original and Scrambled groups. Additionally, we compared the identity effect (within-between) across the two groups for each core face-selective regions, only the right and left FFA demonstrated a consistent identity effect across both the Original and Scrambled groups. None of the core regions showed significant differences in identity representation between the Original and Scrambled groups. A consistent identity effect across the Original and Scrambled groups was observed in several regions identified through the ISC analysis. These regions included the accumbens, amygdala, insula, posterior cingulate, postcentral gyrus, precentral gyrus, and supramarginal gyrus.

**Table 3.** Identity-specific representation results for core face-selective areas (OFA, occipital face area; FFA, fusiform face area; STS, superior temporal sulcus)

Region	Hemisphere	Original (within > between)			Scrambled (within > between)		
		Δ*z*	*t*	*p*	Δ*z*	*t*	*p*
OFA	L	0.19	1.91	0.108	0.13	1.86	0.144
	R	0.02	0.22	0.897	0.02	0.35	0.727
FFA	L	**0.40**	**3.35**	**0.013**	**0.30**	**3.89**	**0.006**
	R	**0.37**	**3.26**	**0.013**	**0.26**	**3.05**	**0.021**
STS	L	0.09	0.77	0.538	0.17	1.68	0.144
	R	0.23	2.36	0.059	0.18	1.63	0.144

Δ*Z* denotes mean difference in correlation (Fisher's *z*) value of within-identity versus between-identity. *p* values are corrected for multiple comparisons. Significant contrasts are highlighted in bold.

**Table 4.** Identity-specific representation results for extended network of regions found in the ISC analysis

Region	Hemisphere	Original (within > between)			Scrambled (within > between)		
		Δ*z*	*t*	*p*	Δ*z*	*t*	*p*
Accumbens	L	0.25	2.46	0.058	**0.25**	**2.76**	**0.033**
	R	**0.29**	**2.69**	**0.047**	**0.27**	**3.15**	**0.024**
Amygdala	L	0.25	2.32	0.062	**0.37**	**5.10**	**0.001**
Anterior cingulate	R	0.19	1.98	0.103	0.12	1.20	0.315
Frontal pole	L	0.30	2.26	0.066	0.00	0.04	0.970
	R	0.20	1.50	0.208	0.02	0.23	0.875
Insula	R	**0.35**	**4.87**	**0.002**	**0.23**	**2.79**	**0.033**
Mid cingulate	L	0.16	1.48	0.208	**0.25**	**2.96**	**0.033**
	R	0.09	0.75	0.513	0.14	1.23	0.315
Medial prefrontal cortex	L	0.24	2.08	0.090	0.17	2.30	0.070
	R	0.05	0.37	0.738	0.13	1.38	0.272
Posterior cingulate	L	**0.28**	**2.76**	**0.047**	**0.25**	**3.41**	**0.016**
	R	0.28	2.48	0.058	0.24	2.21	0.078
Postcentral gyrus	L	**0.25**	**2.68**	**0.047**	**0.21**	**2.76**	**0.033**
	R	**0.46**	**3.17**	0.028	0.06	0.47	0.710
Postcentral gyrus 2	R	0.20	2.50	0.058	**0.27**	**2.56**	**0.043**
Precentral gyrus	L	**0.39**	**3.98**	0.009	**0.27**	**2.83**	**0.033**
	R	**0.46**	**2.92**	0.041	0.26	1.98	0.116
Precentral gyrus 2	R	0.14	1.22	0.295	**0.40**	**4.41**	**0.003**
Precuneus	L	0.14	1.11	0.334	0.02	0.17	0.900
	R	0.12	0.96	0.401	0.08	0.50	0.710
Precuneus 2	L	−0.03	−0.19	0.851	0.10	1.15	0.330
	R	0.07	0.42	0.726	0.17	1.23	0.315
Superior frontal gyrus	L	**0.60**	**5.28**	0.002	0.29	1.88	0.131
	R	**0.37**	**3.66**	0.014	0.09	0.85	0.487
Superior parietal lobule	R	0.24	1.81	0.128	**0.43**	**4.97**	**0.001**
Supramarginal	L	0.19	1.46	0.208	0.21	1.68	0.170
	R	0.26	2.35	0.062	**0.42**	**5.82**	**0.001**
Supramarginal 2	R	0.19	2.37	0.062	**0.40**	**3.71**	**0.010**
Temporal parietal junction	L	0.30	1.83	0.128	**0.25**	**2.66**	**0.038**
	R	**0.32**	**3.36**	**0.022**	0.15	1.74	0.162

Δ*Z* denotes mean difference in correlation (Fisher's *z*) value. *p* values are corrected for multiple comparisons. Significant contrasts are highlighted in bold.

A central assumption underlying the interpretation of the MVPA in the face-selective regions is that the neural representations primarily encode character identity. However, without evaluating responses in early visual areas, it remains unclear to what extent these representations reflect high-level identity information as opposed to low-level perceptual similarity. To disentangle these contributions, we conducted control analyses in two additional regions: (1) an early visual ROI comprising a combined V1–V3 mask based on group-level visual field maps (Wang et al., 2015) and (2) a high-level, non-face visual ROI encompassing place-selective regions (OPA, PPA, and RSC), which respond robustly to scenes but not to faces.

In the early visual cortex (V1–V3), within-identity pattern similarity was significantly greater than between-identity similarity for both the Original (*t*_(18)_ = 6.1, *p* < 0.001) and Scrambled (*t*_(18)_ = 3.3, *p* < 0.001) groups, though the difference between groups was not statistically significant (*t*_(35.8)_ = 1.73, *p* = 0.092). These findings indicate that perceptual similarity alone can drive above-chance identity decoding in early visual areas. In contrast, in the non-face, place-selective control regions, within-identity correlations did not exceed between-identity correlations in either the Original (*t*_(18)_ = 1.89, *p* = 0.075) or Scrambled (*t*_(18)_ = 1.56, *p* = 0.135) groups, and no significant group differences were observed (*t*_(28.9)_ = 0.85, *p* = 0.404). This pattern suggests that while perceptual similarity contributes to identity-related effects at early stages of processing, such effects are not ubiquitous across high-level visual cortex and appear more selective to face-processing systems.

To further dissociate perceptual and conceptual contributions to multivoxel pattern representations, we repeated the MVPA in core and extended face regions only using the characters (Annie, Chris, and Ray) who did not feature prominently in the encoding video. If identity effects in these regions are primarily perceptual, within-identity correlations should exceed between-identity correlations regardless of prior exposure. However, a three-way repeated-measures ANOVA revealed no significant main effects of Identity (*F*_(1,36)_ = 1.23, *p* = 0.275, *η*^2^ = 0.03), Group (*F*_(1,36)_ = 0.18, *p* = 0.677, *η*^2^ = 0.01), or Region (*F*_(1,36)_ = 0.40, *p* = 0.532, *η*^2^ = 0.01) and no significant interactions (all *p* > 0.05). These results suggest that identity-related effects in core and extended face-selective regions are not driven solely by perceptual similarity but instead rely on the presence of conceptual or episodic associations formed during encoding.

## Discussion

Face recognition plays a crucial role in social interaction, yet the neural mechanisms underlying how we become familiar with faces are not fully understood. While existing research has emphasized the role of perceptual experience, face recognition in real-world contexts also involves the integration of conceptual information about the person. In the present study, participants viewed an encoding movie from the TV series *Life on Mars*. This enabled faces to be encountered in a dynamic, context-rich environment, in which the integration of conceptual knowledge about individuals could occur within an evolving narrative. Conceptual information was manipulated by presenting the movie to participants in either its original sequence or a scrambled sequence. Both groups were exposed to the same perceptual input, yet this manipulation had a significant effect on the conceptual understanding of events. Consistent with previous work (Noad and Andrews, 2024), we demonstrated that participants who viewed the original coherent sequence of the movie exhibited better conceptual understanding of the narrative and enhanced face recognition, persisting for several weeks. Furthermore, enhanced conceptual knowledge was associated with increased intersubject correlations of neural responses in many non-visual regions of the brain.

Memory consolidation is widely theorized to depend on the progressive strengthening of connections between distributed neocortical representations and the hippocampus (Squire and Zola-Morgan, 1991; Nadel and Moscovitch, 1997; Yonelinas et al., 2019). This process is facilitated when information is encoded within a coherent contextual framework (Van Kesteren et al., 2010; Lewis and Durrant, 2011) or when deeper, semantically rich processing occurs at the time of encoding (Craik and Lockhart, 1972; Bower and Karlin, 1974). These insights form the basis of the levels-of-processing framework (Craik, 2002), which emphasizes the role of conceptual analysis in shaping long-term memory representations. However, the specific neural mechanisms by which such semantic elaboration influences memory encoding remain unresolved. One possibility is that conceptual knowledge enhances perceptual encoding, thereby strengthening stimulus-specific representations in modality-selective regions such as the core face-processing network (Winograd, 1981; Oh et al., 2021). An alternative account posits that semantic knowledge engages higher-order, non-visual cortical systems associated with person-specific information (Haxby et al., 2000; Gobbini and Haxby, 2007; Ishai, 2008; Kovács, 2020). The current findings support this latter view. Face recognition performance was enhanced when individuals possessed rich conceptual knowledge about the characters, and this effect was accompanied by increased engagement of socially relevant, non-perceptual brain regions. These results align with recent evidence demonstrating that social-evaluative encoding tasks amplify recognition and preferentially recruit extended person-knowledge networks, when compared with purely perceptual encoding tasks (Shoham et al., 2022).

To investigate the effect of conceptual information on neural responses to faces, participants viewed a recognition movie featuring the faces of the main characters after a delay of ∼4 weeks, while brain activity was measured using fMRI. To assess how narrative coherence during encoding modulates neuronal activity to faces during the recognition movie, we measured the similarity in response across the brain between participants in each group using intersubject correlation (ISC). Differences in ISC between the Original and Scrambled groups were found within a network of regions beyond visual cortex which have been previously implicated in familiar face processing (Haxby et al., 2000; Gobbini and Haxby, 2007; Ishai, 2008; Kovács, 2020; Noad et al., 2024). For example, higher ISCs were found in the Original group in regions which are associated with theory of mind (Frith and Frith, 1999) and the perception of personality traits (Gobbini et al., 2004; Visconti di Oleggio Castello et al., 2017; Raykov et al., 2021), such as the temporoparietal junction, anterior cingulate, and medial prefrontal cortex. A similar modulation of ISC by conceptual knowledge was observed in the posterior cingulate and precuneus, which have previously been linked to memory retrieval for faces (Rugg et al., 2002; Dickerson and Eichenbaum, 2010; Silson et al., 2019), and the representation of person knowledge (Thornton and Mitchell, 2017; Afzalian and Rajimehr, 2021; Ragni et al., 2021). Additionally, neural responses in the insula, accumbens, and amygdala were influenced by conceptual knowledge, likely reflecting affective responses to familiar faces (Gobbini et al., 2004; Harris et al., 2012; Ramon and Gobbini, 2018).

In contrast to our findings in non-visual brain regions, we did not observe any significant effect of conceptual understanding within the core face-selective regions. These regions are primarily involved in the visual representation of faces, yet their precise role in face recognition remains a matter of debate. Although models of face recognition (Haxby et al., 2000) have suggested the involvement of the FFA in distinguishing familiar from unfamiliar faces, the evidence is inconsistent. A number of studies have reported no significant differences in FFA activation when participants view familiar compared with unfamiliar faces (Leveroni et al., 2000; Gorno-Tempini and Price, 2001; Gobbini et al., 2004; Davies-Thompson et al., 2009, 2013). Moreover, even when familiarity effects are observed in the FFA, these effects tend to be modest in magnitude (Sergent et al., 1992; Ewbank and Andrews, 2008; Andrews et al., 2010; Axelrod and Yovel, 2015; Weibert and Andrews, 2015). This inconsistency suggests that the core face-selective regions may not be sufficient for the recognition of familiar faces.

In contrast to prior studies that explicitly manipulated or assessed conceptual processing during face encoding (Bower and Karlin, 1974; Patterson and Baddeley, 1977; Schwartz and Yovel, 2016, 2019), the present study adopted a more naturalistic design, omitting explicit tasks during both the encoding and recognition phases. Participants engaged with dynamic social content in a manner that more closely mirrors real-world face perception, including unconstrained eye movements. This design raises the possibility that variability in gaze behavior across participants could reduce intersubject correlation (ISC) in visually driven cortical regions, thereby attenuating observable group-level effects in these areas. Specifically, one might expect lower ISC in visual compared with non-visual regions, which could account for group differences emerging more prominently in extended (conceptual) rather than core (perceptual) face-processing areas. To test this possibility, we directly compared ISC (collapsed across groups) in early visual cortex, core face regions, and extended face regions. Contrary to the hypothesis that visual variability would reduce synchrony, ISC was significantly higher in early visual regions and the core face regions compared with the extended face network. These findings align with prior research demonstrating that eye movements during naturalistic movie viewing are highly consistent across individuals (Hassonet al., et al., 2008; Shepherd et al., 2010; Wang et al., 2012). Moreover, this consistency is preserved even under substantial task or stimulus manipulations, such as temporal scrambling of movie segments or changes in prior contextual knowledge (Wang et al., 2012; Hutson et al., 2017). Thus, our results suggest that gaze-related variability is unlikely to account for the absence of group-level effects in core face regions and instead point toward differences in higher-order processing as the primary source of neural divergence between groups.

Prior research has demonstrated that understanding a narrative can increase the similarity of neural responses across individuals (Hasson et al., 2008; Nguyen et al., 2019; Jääskeläinen et al., 2021). Moreover, manipulating narrative coherence during movie watching has been shown to affect the similarity of neural responses in non-sensory regions (Hasson et al., 2008, 2010; Van Kesteren et al., 2010; Baldassano et al., 2018; Noad et al., 2024). This suggests that participants in the Original group exhibited more synchronized neural activity during the encoding movie due to their exposure to a coherent narrative, which in turn facilitated the consolidation of person knowledge about the characters. In contrast, the recognition phase of our experiment involved a movie that was not narrative driven and was identical for both groups. The segments shown during this phase were drawn from a variety of episodes from the TV series which had not been previously seen. So, while these segments were selected to provide clear views of the characters’ faces, there was no coherent storyline to follow. This key feature of our design allowed us to isolate differences in neural processing specifically related to face perception and recognition during this phase of the experiment.

Contemporary cognitive models of face recognition posit a transformation from image-dependent perceptual codes to abstract, image-invariant representations that support recognition of familiar individuals (Bruce and Young, 1986; Burton et al., 1999; Hancock et al., 2000; Young and Burton, 2017). These invariant representations are believed to form the neural basis of the subjective experience of face familiarity. To examine whether regions within the face-processing network exhibit such image-invariant coding, we assessed multivoxel pattern similarity for individual identities across visually distinct scenes in a naturalistic recognition context (Milivojevic et al., 2016; Lally et al., 2023). Identity-specific coding was operationalized as greater pattern similarity for different instances of the same identity compared with instances of different identities. Robust identity-specific patterns were observed not only in the core face-selective regions (e.g., FFA) but also across components of the extended face network. These findings converge with prior studies demonstrating reliable decoding of familiar face identity within both perceptual and conceptual components of the face network (Natu et al., 2010; Nestor et al., 2011; Anzellotti et al., 2014; Axelrod and Yovel, 2015; Visconti di Oleggio Castello et al., 2017, 2021; Tsantani et al., 2021). While identity decoding in core perceptual regions may, in part, reflect low-level visual similarity—as supported by analogous findings in early visual cortex—identity-specific representations in non-visual, socially, and semantically enriched regions of the extended network suggest a more distributed and conceptual basis for familiar face recognition. These findings underscore the critical role of non-perceptual systems in supporting invariant representations of identity, enabling stable recognition of familiar individuals across changes in visual appearance and environmental context.

Although intersubject correlation (ISC) and multivoxel pattern analysis (MVPA) differ substantially in their methodological approach—one capturing shared temporal dynamics across individuals and the other measuring spatially distributed representations—we observed convergent patterns across these analyses. Specifically, ISC revealed robust group-level differences between the Original and Scrambled conditions within the extended face network, implicating non-visual regions in the encoding of socially and conceptually enriched face information. Consistent with this, MVPA revealed numerically greater identity-specific pattern similarity in the Original group relative to the Scrambled group within extended, but not core, face regions, although these effects did not reach statistical significance. These converging patterns suggest that both ISC and MVPA may be sensitive to common underlying representational processes related to person-specific information. However, ISC appeared to be the more sensitive of the two methods in the context of naturalistic, time-varying stimuli—potentially due to its capacity to capture dynamic, temporally structured neural responses that MVPA (which is typically applied to more controlled, trial-based designs) may fail to detect with equal sensitivity. Together, these findings highlight the utility of combining temporally and spatially resolved analytical approaches to elucidate the neural mechanisms supporting face recognition in ecologically valid settings.

In conclusion, this study investigated the role of conceptual knowledge in processing familiar faces under naturalistic conditions. Our findings demonstrate that conceptual knowledge modulates neural responses to faces in an extended network of regions beyond the core face-selective areas. Additionally, patterns of neural activity within this extended network were able to discriminate between different face identities. These results suggest that non-visual brain regions play a significant role in the recognition of familiar individuals and that conceptual knowledge is a critical component in the processing of familiar faces.
